# Juvenile Dystonia Associated with Heterozygous Missense Variant in *KCNJ10*

**DOI:** 10.1002/mds.30298

**Published:** 2025-07-31

**Authors:** Claudio M. de de Gusmao, Sara C.B. Casagrande, Matheus A. Castro, André Pessoa, Cleonisio Leite Rodrigues, Laura Silveira Moriyama, Fernando Kok, Paulo Ribeiro Nóbrega

**Affiliations:** ^1^ Department of Neurology University of Sao Paulo Sao Paulo Brazil; ^2^ Department of Neurology Universidade Estadual de Campinas (UNICAMP) Campinas Brazil; ^3^ DSMCA Department Federal University of Ceara–UFC Fortaleza Brazil; ^4^ Department of Neurology, Division of Clinical Neurophysiology Hospital Geral de Fortaleza Fortaleza Brazil; ^5^ Institute of Neurology Teaching Unit, UCL Queen Square London United Kingdom; ^6^ Division of Neurology, Department of Clinical Medicine Federal University of Ceará–UFC Fortaleza Brazil

We have read with interest the report by Huang et al describing pathogenic monoallelic variants in the *KCNJ10* gene associated with paroxysmal kinesigenic dyskinesia (PKD).^1^ Here, we report on an individual with segmental dystonia and a pathogenic variant in *KCNJ10*, potentially expanding the phenotypic spectrum.

The proband is a 16‐year‐old boy without a significant past medical history. Around age 14, he developed involuntary right‐hand movements. On examination, there is dystonic posturing of the right hand with wrist flexion, intermittent closure of the fingers, and thumb extension. There are mild dystonic features elsewhere, including mild dystonic posturing of the shoulders and mouth. There was no reported paroxysmal worsening; repeated kinesigenic provocative maneuvers performed in the office were negative (knee‐high stress test) (Video 1). The remainder of the neurological examination was normal, including cognition, hearing, and cerebellar function. MR and magnetic resonance angiography (MRA) imaging of the brain were unremarkable. Electromyography (EMG) demonstrated dystonic muscular activity, with co‐contraction of wrist flexors and extensors and intrinsic hand muscles, worsening during a writing task. Family history was notable for generalized anxiety and borderline personality in his father; mother is healthy. He has a younger sister with attention‐deficit disorder. Laboratory work‐up did not demonstrate electrolyte abnormalities. A treatment trial with levodopa was unsuccessful. Exome sequencing performed in a clinical diagnostic laboratory identified a heterozygous pathogenic variant in *KCNJ10*: NM_002241:c.596G>A, p.Arg199Gln. This variant has been previously reported in the context of paroxysmal dyskinesia and has a functional study determining deleterious impact in protein function.^2^ After diagnosis, carbamazepine was trialed but unsuccessful. We were not able to genotype his parents to determine whether it was inherited or occurred “de novo”.

**Video 1:** Segment 1: dystonic posture of the right upper extremity is seen. There is dystonic slowness on repetitive movements of the right hand, and writing is laborious. Segment 2: there is slight asymmetric shoulder elevation, otherwise gait is unremarkable. A test for paroxysmal kinesigenic dyskinesia (knee‐high test) is negative.

The *KCNJ10* gene encodes the Kir4.1 inwardly rectifying potassium channel, which is important for glial function, control of neuronal excitability, and systemic potassium homeostasis.^3^ In the cases described thus far with *KCNJ10* monoallelic pathogenic variants, the clinical presentation consisted of brief attacks (<10 seconds in most) with predominant dystonic posturing, often affecting the face and/or limbs.^1^, ^2^, ^4^, ^5^ The condition has variable penetrance and good response to carbamazepine.^2^ Previously, biallelic loss‐of‐function variants in *KCNJ10* had been associated with SESAME syndrome (OMIM # 612780), which combines seizures, sensorineural deafness, ataxia, intellectual disability, and electrolyte imbalance due to renal tubulopathy.^6^ Notably, in some reported individuals with SESAME syndrome, dystonia was described in the upper limbs, emerging over time and in the context of additional neurological findings.^7^ Conversely, some individuals with monoallelic variants in *KCNJ10* had subtle signs of SESAME syndrome, such as discrete oculomotor cerebellar findings and renal tubulopathy.^4^ In our patient, dystonia was the only finding. Furthermore, we ascertained that the *KCNJ10* gene was 100% covered, and there were no other variants of interest in this gene or elsewhere in the genome. Although we cannot predict whether additional symptoms will emerge over time in this patient, the stability of the condition for the past few years is reassuring.

*KCNJ10* loss‐of‐function causes neuronal hyperexcitability, putatively causing PKD through abnormal firing of cerebellar cells.^1^ In light of converging evidence linking cerebellar dysfunction and dystonia, we hypothesize that pathogenic monoallelic variants in *KCNJ10* may present in a symptom spectrum and may be also possibly associated with non‐paroxysmal symptoms. Presently, *KCNJ10* mutations are most commonly screened in commercially available gene panels for renal disorders, hearing loss, epilepsy, and ataxia. We suggest *KCNJ10* might be included in research panels for dystonia. Further studies are needed to determine if *KCNJ10* is indeed associated with focal‐onset dystonia in childhood.

## Author Roles

(1) Research Project: A. Conception, B. Organization, C. Execution; (2) Statistical Analysis: A. Design, B. Execution, C. Review and Critique; (3) Manuscript Preparation: A. Writing of the First Draft, B. Review and Critique.

C.M.de G.: 1A, 1B, 1C, 3A

S.C.B.C.: 1B, 1C, 3B

M.A.C.: 1B, 1C, 3B

A.P.: 1C, 3B

C.L.R.: 1C

L.S.M.: 1B, 1C, 3B

F.K.: 3B

P.R.N.: 1C, 3B

## Financial Disclosures of All Authors (for the Preceding 12 Months)

Claudio M. de Gusmao was previously an employee of Mendelics Genomic Analysis until 2024. He has been a speaker for PTC therapeutics. He is a coinvestigator in NIH Fogarty grant R21NS139341. Sara C. B. Casagrande is an employee of the University of Sao Paulo. Matheus A. Castro is currently an employee of Mendelics Genomic Analysis. He is a coinvestigator in NIH Fogarty grant R21NS139341. André Pessoa is currently an employee of Universidade Federal do Ceará (UFC). He does not report any additional financial disclosures. Cleonisio Leite Rodrigues is currently an employee of Hospital Geral de Fortaleza (HGF) and has received speaker fees and consulting honoraria from PTC, Sanofi, and AstraZeneca. Laura Silveira Moriyama is currently an employee of Universidade Estadual de Campinas (UNICAMP), has received research support from Samsung Electronics and SAS Brazil, and small grants/honoraria from Ipsen, FQM‐Roche, AbbVie, Zambon, Merz, International Parkinson, and Movement Disorders Society. Fernando Kok is an employee of Mendelics Genomic Analysis and University of Sao Paulo. He is the Brazilian principal investigator of NIH Fogarty grant R21NS139341. Paulo Ribeiro Nóbrega is currently an employee of Universidade Federal do Ceará (UFC), has received research support from Travere Pharmaceutics, unrelated to his current work, and small grants/honoraria from FQM‐Roche. Cleonisio Leite Rodrigues is an employee of Universidade Federal do Ceará (UFC).

## Data Availability

The data that support the findings of this study are available on request from the corresponding author. The data are not publicly available due to privacy or ethical restrictions.

## References

[1] Huang X , Fu X , Wu J , et al. Heterozygous KCNJ10 Variants Affecting Kir4.1 Channel Cause Paroxysmal Kinesigenic Dyskinesia. Movement Disorders 2024;39(12):2199–2210. 39367724 10.1002/mds.30025

[2] Li Y , Lin J , Huang X , et al. Heterozygous Variants in KCNJ10 Cause Paroxysmal Kinesigenic Dyskinesia Via Haploinsufficiency. Ann Neurol 2024;96(4):758–773. 38979912 10.1002/ana.27018

[3] Sala‐Rabanal M , Kucheryavykh LY , Skatchkov SN , et al. Molecular mechanisms of EAST/SeSAME syndrome mutations in Kir4.1 (KCNJ10). J Biol Chem 2010;285(46):36040–36048. 20807765 10.1074/jbc.M110.163170 PMC2975226

[4] Wirth T , Roze E , Delvallée C , et al. Rare Missense Variants in *KCNJ10* Are Associated with Paroxysmal Kinesigenic Dyskinesia. Mov Disord 2024;39(5):897–905. 38436103 10.1002/mds.29752

[5] Zorzi G , Zibordi F , Sorrentino U , et al. Potassium Channel Subunit Kir4.1 Mutated in Paroxysmal Kinesigenic Dyskinesia: Screening of an Italian Cohort. Movement Disorders 2024;39(12):2302–2304. 39206934 10.1002/mds.30008 PMC11657011

[6] Scholl UI , Choi M , Liu T , et al. Seizures, sensorineural deafness, ataxia, mental retardation, and electrolyte imbalance (SeSAME syndrome) caused by mutations in KCNJ10. Proceedings of the National Academy of Sciences 2009;106(14):5842–5847. 10.1073/pnas.0901749106 PMC2656559 19289823

[7] Cross JH , Arora R , Heckemann RA , et al. Neurological features of epilepsy, ataxia, sensorineural deafness, tubulopathy syndrome. Dev Med Child Neurol 2013;55(9):846–856. 23924083 10.1111/dmcn.12171 PMC4298033

